# Cortical connectivity of the nucleus basalis of Meynert in Parkinson’s disease and Lewy body dementias

**DOI:** 10.1093/brain/awaa411

**Published:** 2020-12-26

**Authors:** Ashwini Oswal, James Gratwicke, Harith Akram, Marjan Jahanshahi, Laszlo Zaborszky, Peter Brown, Marwan Hariz, Ludvic Zrinzo, Tom Foltynie, Vladimir Litvak

**Affiliations:** 1 Medical Research Council Brain Network Dynamics Unit, University of Oxford, Oxford, UK; 2 Nuffield Department of Clinical Neurosciences, John Radcliffe Hospital, Oxford, UK; 3 Wellcome Centre for Human Neuroimaging, UCL Institute of Neurology, London, UK; 4 Department of Clinical and Movement Neurosciences, UCL Institute of Neurology and The National Hospital for Neurology and Neurosurgery, Queen Square, London, UK; 5 Center for Molecular and Behavioral Neuroscience, Rutgers University, Newark, USA; 6 Department of Clinical Neuroscience, Umeå University, Umeå, Sweden

**Keywords:** DBS, MEG, DTI, coherence, oscillations

## Abstract

Parkinson’s disease dementia (PDD) and dementia with Lewy bodies (DLB) are related conditions that are associated with cholinergic system dysfunction. Dysfunction of the nucleus basalis of Meynert (NBM), a basal forebrain structure that provides the dominant source of cortical cholinergic innervation, has been implicated in the pathogenesis of both PDD and DLB. Here we leverage the temporal resolution of magnetoencephalography with the spatial resolution of MRI tractography to explore the intersection of functional and structural connectivity of the NBM in a unique cohort of PDD and DLB patients undergoing deep brain stimulation of this structure. We observe that NBM-cortical structural and functional connectivity correlate within spatially and spectrally segregated networks including: (i) a beta band network to supplementary motor area, where activity in this region was found to drive activity in the NBM; (ii) a delta/theta band network to medial temporal lobe structures encompassing the parahippocampal gyrus; and (iii) a delta/theta band network to visual areas including lingual gyrus. These findings reveal functional networks of the NBM that are likely to subserve important roles in motor control, memory and visual function, respectively. Furthermore, they motivate future studies aimed at disentangling network contribution to disease phenotype.

## Teaser

Oswal *et al.* study the structural and functional connectivity of the nucleus basalis of Meynert (NBM) in patients with PDD and DLB. By integrating NBM recordings, MEG and MRI tractography, they identify frequency-specific pathophysiological networks where functional connectivity is predicted by structural connectivity.

## Introduction

Parkinson’s disease dementia (PDD) and dementia with Lewy bodies (DLB) are two of the commonest neurodegenerative dementias (Aarsland and Kurz, 2010; Jellinger, 2018). They are both characterized by the neuropathological hallmark of cortical Lewy bodies composed of alpha-synuclein (Hurtig *et al.*, 2000; Horvath *et al.*, 2013) and are associated with marked cholinergic neurotransmitter system dysfunction (Shimada *et al.*, 2009). They also share a common phenotype including prominent executive, attentional and visual processing dysfunction, memory deficits, cognitive fluctuations, visual hallucinations and parkinsonism (Emre *et al.*, 2007; McKeith *et al.*, 2017). Clinical differentiation between these two interrelated conditions depends on whether dementia occurs in the context of established Parkinson’s disease (PDD) or prior to/concurrent with parkinsonian symptoms (DLB).

The nucleus basalis of Meynert (NBM) is the principal source of cholinergic inputs to cortex, which are implicated in memory, attention, visual processing and motor plasticity (Mesulam and Geula, 1988; Gratwicke *et al.*, 2013). Furthermore, degeneration of the NBM has been shown to predict cognitive impairments in both PDD and DLB (Whitehouse *et al.*, 1983; Choi *et al.*, 2012; Grothe *et al.*, 2014; Ray *et al.*, 2018). Strategies aimed at modulating the activity of the NBM and its cortical efferents have been proposed as therapies for both conditions (Gratwicke *et al.*, 2013). We have recently trialled deep brain stimulation (DBS) of the NBM as a potential therapy for both PDD and DLB (Gratwicke *et al.*, 2018, 2020*b*). Our studies revealed that low frequency stimulation (20 Hz) of the NBM can be successfully and safely carried out in both PDD and DLB with the potential for possible improvements in neuropsychiatric symptoms (Gratwicke *et al.*, 2018, 2020*b*). These studies have simultaneously offered the opportunity to gain unique neurophysiological insights into the function of the NBM and its cortical networks. By combining recordings of NBM local field potentials (LFP) from DBS electrodes with magnetoencephalography (MEG), we have been able to identify NBM-cortical networks that are common to both diseases (Gratwicke *et al.*, 2020*a*) including: (i) a delta/theta band (2–8 Hz) network between the NBM and temporal cortex; and (ii) a low beta band (13–22 Hz) network between the NBM and mesial motor areas. Despite this, it remains unclear how the functional connectivity of the NBM relates to its structural connectivity.

Previous work with MRI tractography reveals extensive structural projections of the NBM to cortical regions (Hepp *et al.*, 2017; Nemy *et al.*, 2020). It is likely, however, that the outputs of the basal forebrain structures are both spatially and temporally (Gielow and Zaborszky, 2017; Záborszky *et al.*, 2018) segregated representing discrete functional networks that subserve distinct roles. An improved understanding of the segregation of these networks is essential for understanding their functional and pathophysiological roles.

Here we test for spatiotemporal segregation of NBM-cortical networks, by integrating previously reported (Gratwicke *et al.*, 2020*a*) MEG and NBM LFP recordings with MRI tractography derived from open source connectomes (Horn *et al.*, 2017). Using this approach, we test for the presence of brain networks that are both structurally and functionally connected to the NBM.

## Materials and methods

Six patients with PDD and five patients with DLB who participated in the clinical trials underwent combined MEG and NBM LFP recordings. Clinical characteristics of the patients and details of the surgical procedure are provided in previous clinical and MEG studies of the same cohort (Gratwicke *et al.*, 2018, 2020*a*, *b*). Study procedures were approved by the East of England Research Ethics Service Committee, and the patients gave written informed consent prior to participation.

**Figure 1:**
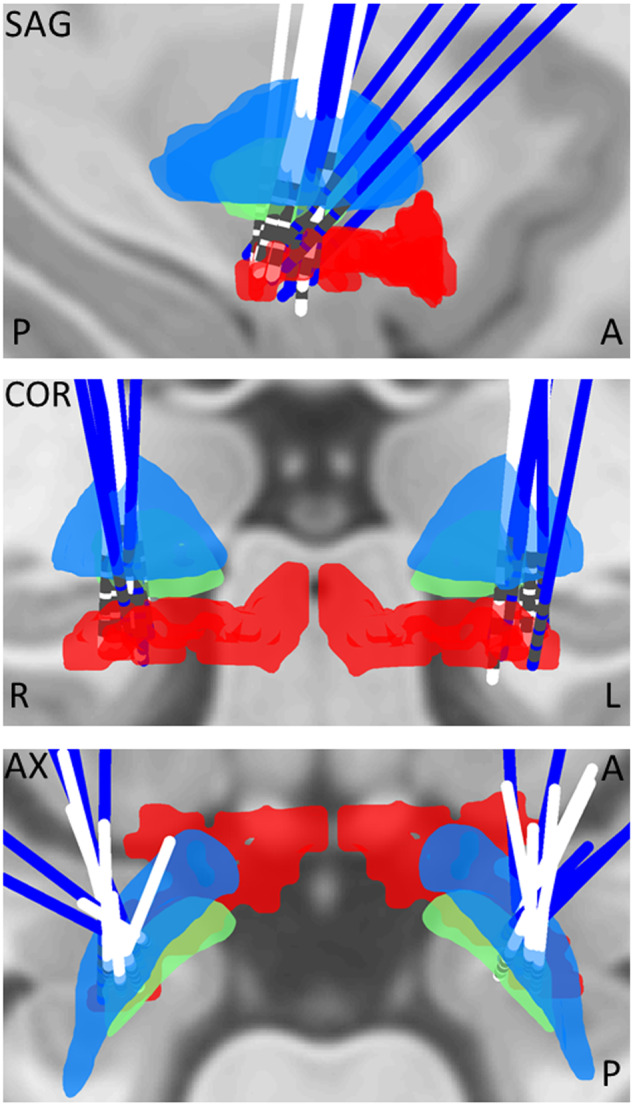
**Localization of electrode trajectories and contacts in MNI space.** Electrodes in the DLB cohort are coloured in dark blue whilst electrodes in the PDD cohort are coloured white. Templates of GPi (green), GPe (light blue) and basal forebrain (red), which includes the NBM are superimposed on a T_1_-weighted structural MRI for visualization. The 3D image is viewed in the axial, sagittal and coronal planes. A = anterior; Ax = axial; COR = coronal; L = left; P = posterior; R = right; SAG = sagittal.

Contact localization of the bilateral quadripolar Medtronic electrodes was confirmed postoperatively with stereotactic MRI (proton density sequence) and visualized using the Schaltenbrand atlas (Gratwicke *et al.*, 2018, 2020*b*). The most ventral contact (contact 0) was successfully placed in the Ch4i sector of the NBM in all patients. Figure 1 shows electrode and contact locations for the PDD (white) and DLB (blue) patient groups in MNI (Montreal Neurological Institute) template space generated using Lead-DBS software (Horn and Kühn, 2015; Horn *et al.*, 2019). Outlines of the internal and external globus pallidus (GPi and GPe) generated using the DISTAL atlas within Lead-DBS are also displayed in addition to an outline of the basal forebrain (Teipel *et al.*, 2005). In keeping with the results from individual anatomy contact 0 can be seen to be well positioned within the NBM across subjects.

The second most ventral contact (contact 1) in contrast was most frequently located either in the NBM or in the border between the NBM and the GPi. There were, however, five electrodes (of a total of 22 electrodes) from three patients (four electrodes from two patients with PDD and one electrode from a patient with DLB) where contact 1 skimmed a portion of the inferior GPi. As the primary focus of this study was on exploring structural and functional connectivity patterns of the NBM, we excluded these electrodes from further analysis.

In the PDD cohort an additional aim of electrode placement was to target the upper contacts (2 and 3) to the posteroventral GPi. This allowed for the future possibility of stimulation of the GPi to aid the motor symptoms of PDD. Targeting of the posteroventral GPi was deemed to be less clinically important in the DLB cohort, however, due to lower motor symptom severity in these patients. Owing to this, in the DLB cohort we were able to select a target for the deepest contacts (0 and 1) that was in the thickest and widest part of the Ch4i sector of the NBM that was a few millimetres anteromedial to the target selected for the PDD cohort (Gratwicke *et al.*, 2018, 2020*b*). Thus, in some patients (more commonly in the DLB group), the top contacts were not inside GPi but in the dorsal GPe (Fig. 1). Therefore we refer to the location of the upper contacts 2 and 3 as being in the pallidal (GP) region (Gratwicke *et al.*, 2020*a*). Crucially, the goal of comparing the structural and functional connectivity profiles of contacts 0–1 (representing the NBM) and contacts 2–3 (representing the GP region) was to determine whether any effects observed were specific to the NBM.

### Electrophysiological recordings

MEG recordings were performed using a 275-channel MEG system (CTF/VSM MedTech). LFP activity recorded from DBS electrodes was collected at the same time as MEG using a BrainAmp system (Brain Products). Three bipolar channels (0-1, 1-2, 2-3) were recorded from each electrode and were high-pass filtered at 1 Hz in the hardware to avoid amplifier saturation due to large DC offsets. Rest recordings of a duration of 3 min were performed whilst patients were ON their usual medication.

### Magnetoencephalography and tractography analysis

The aim of joint MEG and LFP analysis was to generate an MNI space whole brain image of coherence—which we used as a measure of frequency specific functional connectivity—between the NBM (contact pair 0-1) or GP region (contact pair 2-3) and 5 mm spaced grid points within the brain. For this purpose we used a single shell forward model, based on each patient’s preoperative MRI (Oswal *et al.*, 2016), in conjunction with Dynamic Imaging of Coherent Sources (DICS) beamforming implemented in the Data Analysis in Source Space (DAiSS) toolbox for SPM12 (https://github.com/spm/DAiSS). Values at the grid points were then interpolated to produce coherence images with 2 mm resolution. Based on previous analysis (Gratwicke *et al.*, 2020*a*) we restricted our analyses of functional connectivity with the NBM to the delta/theta (2–8 Hz), low beta (13–22 Hz) and high beta (22–30 Hz) bands.

Lead-DBS software was used to localize electrode contacts in the aforementioned MNI space as described above. For the purposes of fibre tracking, a spherical region of interest centred at the midpoint of each chosen contact pair, with a radius that just encompassed each contact pair was constructed. Additionally, fibres were restricted to those that also traversed the structure of interest, which was either the NBM for contact pair 0-1 or the GPi for contact pair 2-3. This spherical volume was used as a seed region in an openly available group connectome (www.lead-dbs.org), which was derived from the diffusion-weighted MRIs of 32 healthy subjects within the human connectome project. Whole brain tractography fibre sets were calculated within a white-matter mask after segmentation with SPM12 using DSI studio (http://dsi-studio.labsolver.org). Fibre tracts were transformed into MNI space for visualization. The number of fibres passing through both the spherical seed and each 2 × 2 × 2 mm cubic voxel served as an estimate of tract density and was written to a 3D image. Images corresponding to left LFP channels were flipped across the mid-sagittal plane to allow comparison of ipsilateral and contralateral sources regardless of original side. Tract density images were compared at the group level using a 2 × 2 ANOVA in SPM12 with factors contact location (NBM versus GP region) and disease (PDD versus DLB). We included covariates in order to account for both subject-specific dependencies in the recordings from both hemispheres and for potential differences between recordings of the right and left sides.

### Integration of structural and functional connectivity

We used a group level voxel-wise general linear model (GLM) for determining whether structural connectivity was predictive of cortico-LFP coherence separately for each of the two frequency bands and for NBM and GP region contact pairs. After right-flipping both coherence and tract density images corresponding to left LFP channels, we constructed a GLM for each voxel with tract density as the independent variable and coherence as the dependent variable. This is different from the standard SPM approach since here the design matrix is voxel-specific rather than the same for the whole brain. A cluster-based permutation test with cluster-forming threshold of *P *<* *0.01 was used to define significance. We report results significant at *P *<* *0.01 family-wise error corrected at the cluster level. F-statistics of voxels within each cluster were then written to a 3D nifty format image for visualization.

### Directionality analysis

The effective directionality of coupling between the cortex and the NBM LFP was computed with a non-parametric variant of spectral Granger causality (Dhamala *et al.*, 2008). To determine the significance of directionality estimates, we compared the Granger estimate of original data to that of surrogate time-reversed data using a paired *t*-test. This procedure has proven to suppress the influence of weak data asymmetries not related to time-lagged interactions in the data, while it is statistically powerful in the detection of meaningful strong asymmetries (Haufe *et al.*, 2013). Taking the example of two signals A and B, with A Granger causing B, the Granger causality from A to B should be higher for the original than for the time-reversed data, giving rise to a positive difference. In contrast, the estimate of causality from B to A should be increased by time reversal thereby giving a negative difference.

### Data availability

Exemplar code for computation of structural and functional connectivity relationships can be found on the following GitHub repository (https://github.com/AshOswal/Multimodal_Tools). Anonymized data are available from the corresponding authors on request.

## Results

### Visualization of tracts and oscillatory networks

**Figure 2:**
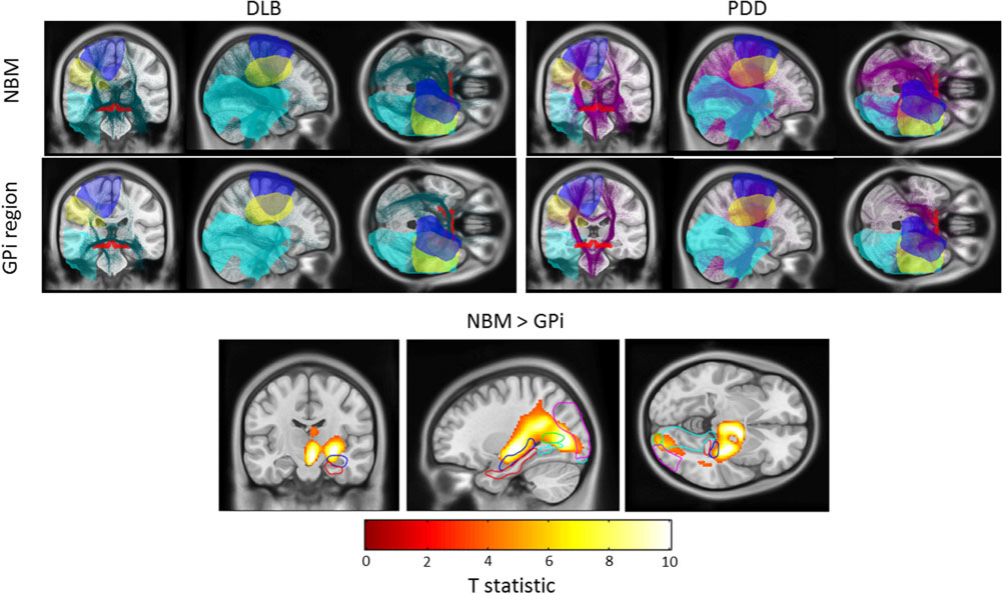
**Visualization of tracts and oscillatory networks.** *Top*: Fibre streamlines passing from the vicinity of NBM and GP region contacts to cortical regions are displayed for the two disease groups on a T_1_-weighted MRI scan. For DLB patients, fibres are coloured green, whilst for PDD patients they are coloured magenta. MEG-derived cortical networks displaying coherence with both the GPi and NBM in the delta/theta (turquoise surface), low beta (blue surface) and high beta (yellow surface) bands are also displayed. *Bottom*: Statistical Parametric Map (SPM) displaying the results of the 2 × 2 ANOVA with factors disease (PDD versus DLB) and contact location (NBM versus GPi). The statistical T-image displays regions that have significantly greater structural connectivity with the NBM than with the GPi (main effect of location). These include the hippocampus (blue contour), lingual gyrus (turquoise contour), calcarine cortex (green contour), and occipital cortex (magenta contour). The parahippocampal gyrus is indicated by the red contour.

Figure 2 (top) displays tractography streamlines of fibres passing from the vicinity of NBM or GP region contacts to cortical structures for the PDD and DLB patient groups. In the case of NBM contacts there are extensive fibre connections to numerous cortical regions (‘corticopetal’ connections). MEG derived cortical networks exhibiting coherence with both the NBM and GP region at delta/theta (2–8 Hz; turquoise), low beta (13–22 Hz; blue) and high beta (22–30 Hz; yellow) frequencies are also displayed. Note that the MEG networks were derived after right flipping of images corresponding to the left LFP and are, therefore, predominantly ipsilateral. Figure 2 (bottom) displays the results of the 2 × 2 ANOVA comparing tract densities with factors contact location (NBM versus GP region) and disease (PDD versus DLB). The only significant finding was a main effect of contact location, such that NBM contacts displayed greater structural connectivity with a region including the hippocampus (blue contour), the lingual gyrus (turquoise contour), the calcarine cortex (green contour), and occipital cortex (magenta contour). Because of the lack of a main effect of disease for both structural and functional connectivity, we pooled across the disease states for further analysis, separately for GP region and NBM contacts.

### Structural connectivity predicts functional connectivity within NBM-cortical networks

**Figure 3:**
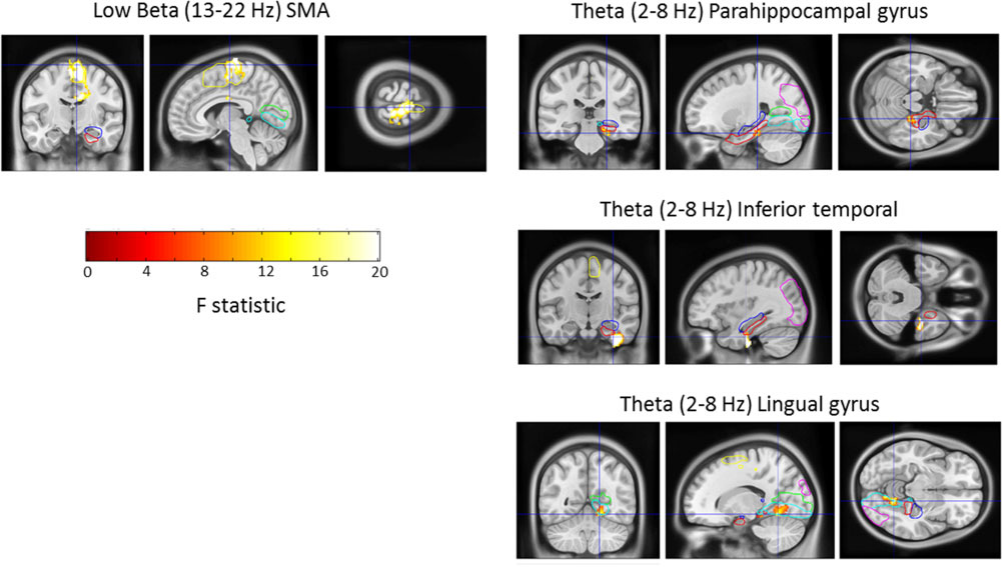
**Spatially and spectrally distinct cortico-NBM networks with correlated structural and functional connectivity.** *Left*: A statistical image showing premotor areas (including SMA; yellow contour) where structural connectivity and coherence with the NBM in the low beta band are correlated. *Right*: Regions where structural connectivity and coherence with the NBM in the delta/theta band are correlated. Three clusters are displayed that include the parahippocampal gyrus, the inferior temporal cortex and the lingual gyrus. Images are superimposed on a T_1_-weighted MRI scan and the colour bar represents the value of the F-statistic. Contours of the supplementary motor area (SMA; yellow contour) hippocampus (blue), parahippocampal gyrus (red), lingual gyrus (turquoise contour), calcarine cortex (green), and occipital cortex (magenta contour) are also shown.

**Figure 4:**
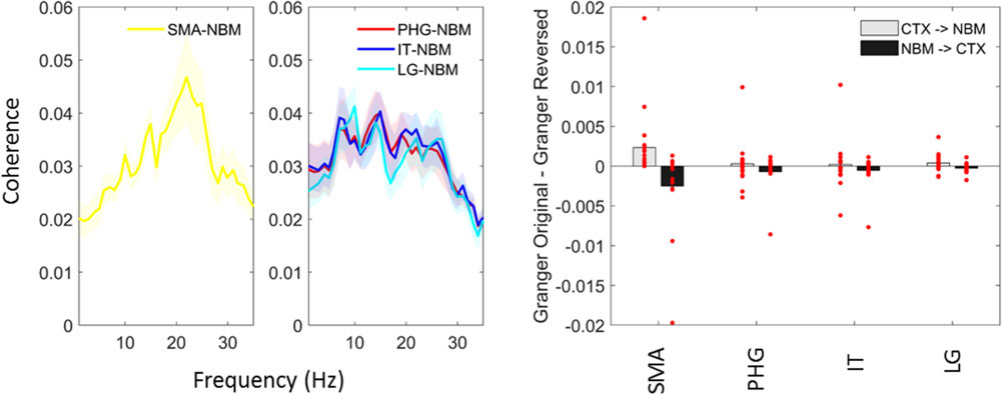
**Group spectra and directionality analysis.** (**A**) *Left* panel shows coherence spectra computed between the NBM LFP and the location of the peak F statistic of the correlation between beta band coherence and tract density which was within the SMA. Similarly, in the *right* panel coherence between the NBM LFP and cortical locations displaying a correlation between structural and functional connectivity in the delta/theta band is plotted. (**B**) Group mean differences in Granger causality between the original data and time reversed data are averaged across the beta band for the SMA and across the delta/theta band for the parahippocampal gyrus (PHG), the inferior temporal cortex (IT) and lingual gyrus (LG). Individual data-points are shown in red. The difference in Granger causality is significantly greater than zero in the direction of SMA leading the NBM in the beta band. CTX = cortex.

Figure 3 (left) reveals cortical regions where NBM-cortical tract density was predictive of NBM-cortical coherence in the low beta frequency range (13–22 Hz). The significant cluster encompasses the SMA. In contrast, Fig. 3 (right) reveals regions where NBM-cortical tract density was predictive of NBM-cortical coherence in the delta/theta frequency range (2–8 Hz). Three clusters are displayed that include the parahippocampal gyrus (red contour), the inferior temporal cortex and the lingual gyrus (turquoise contour). Source extracted coherence spectra for the peak locations in Fig. 3 are shown in the Fig. 4A. These reveal peaks in the beta and delta/theta bands, respectively.

Importantly we have observed differences in the structural connectivity profiles of the NBM and GPi, despite their functional connectivity (Gratwicke *et al.*, 2020*a*) profiles being similar. Furthermore, no significant relationship between GPi region-cortical tract density and GPi region-cortical coherence was observed in any of the three frequency bands.

### Directionality of coupling within structurally and functionally connected cortico-NBM networks

For the purposes of directionality analysis, we derived source time series from the locations of the peak F-statistics of the correlation between structural and functional connectivity (Fig. 3), separately for the delta/theta and beta bands. For the SMA-NBM beta band network, the difference in granger causality, averaged across the beta band, was significantly greater than zero in the direction of SMA leading the NBM [*F*(1,8) = 5.7, *P *=* *0.044; Fig. 4B] suggesting cortical driving of the NBM. In contrast, for the parahippocampal gyrus-NBM [cortex leading *F*(1,8) = 0.13, *P *=* *0.73; NBM leading *F*(1,8) = 1.75, *P *=* *0.22], the inferior temporal-NBM [cortex leading *F*(1,8) = 0.02, *P *=* *0.88; NBM leading *F*(1,8) = 1.02, *P *=* *0.34] and the lingual gyrus-NBM networks [cortex leading *F*(1,8) = 2.26, *P *=* *0.17; NBM leading *F*(1,8) = 2.77, *P *=* *0.13], the difference in granger causality averaged across the delta/theta band was not significant in any particular direction, hence suggesting bidirectional patterns of communication.

## Discussion

In this report we develop a methodology to explore the intersection of structural and functional connectivity and using this we identify distinct brain networks that are both structurally and functionally connected to the NBM in patients with DLB and PDD. Importantly, the correlation of structural and functional connectivity was specific to the NBM rather than to the GP region, which we hypothesize may be reflective of a monosynaptic input-output relationship between the NBM and cortical structures (Saper, 1984; Gielow and Zaborszky, 2017; Záborszky *et al.*, 2018). The GPi/GPe in contrast have polysynaptic cortical connections via intermediate structures (Nambu, 2007) such as the thalamus and other basal ganglia nodes.

We identified three spatially and spectrally distinct NBM-cortical networks with overlapping structural and functional connectivity. First, we identified a low beta band network (13–22 Hz) between the NBM and the SMA, an area known to be important in the volitional control of movement (Nachev *et al.*, 2008). The existence of this network is consistent with previous tracer studies in humans, rodents and primates demonstrating monosynaptic connections projecting from both the NBM to the sensorimotor areas and vice versa (Mesulam and Mufson, 1984; Mesulam and Geula, 1988; Gielow and Zaborszky, 2017). It is hypothesized that such cortico-NBM projections may play important roles in motor plasticity and skill learning. Importantly, within this network we observed that cortical activity tended to drive NBM activity. This direction of information flow appears more consistent with the hypothesized ‘top-down’ model of the fronto-parietal attention network, wherein direct frontal cortical connections to NBM modulate its cholinergic output to other cortical areas to amplify processing of attention demanding signals (Duque *et al.*, 2000; Sarter *et al.*, 2005). Furthermore, modulation of attention within this network has been shown to be related to functional connectivity in the beta band (Buschman and Miller, 2007). Interestingly, in addition to driving NBM activity at low beta frequencies, the SMA also couples with and drives subthalamic nucleus activity at high beta (21–30 Hz) band frequencies which may possibly be reflective of hyperdirect pathway activity (Oswal *et al.*, 2016). These findings, therefore, indicate that the outputs of the SMA to different anatomical structures may be spectrally segregated.

Second, two spatially distinct delta/theta (2–8 Hz) band networks were seen; one including inferior and mesial temporal lobe structures such as the parahippocampal gyrus and a second including lingual gyrus. Based on spatial location, it is likely that these networks may have important roles in memory and visual function, respectively (Gratwicke *et al.*, 2013; Ballinger *et al.*, 2016; Huppé-Gourgues *et al.*, 2018). In contrast to the beta band network, we did not identify a net directionality of information flow within the theta band network. This is likely to reflect bidirectional patterns of communication between the NBM and mesial temporal structures such as the hippocampus and parahippocampal cortex, which have also been demonstrated with tracer studies (Mesulam and Geula, 1988; Gratwicke *et al.*, 2013).

Our findings should be interpreted in light of the limitation that we used tractography data from normative connectomes rather than from individual subjects. Nevertheless, this approach also offers a major advantage in that connectome data are acquired with specialized hardware and large cohort sizes leading to connectivity estimates with improved signal-to-noise ratio compared to what would be possible with individual patient data. Connectome data have been successfully leveraged recently to study the mechanisms of DBS action (Horn *et al.*, 2017) and also to explore how data from disparate lesion studies can be integrated to understand the role of brain networks in disease (Fox, 2018).

In summary, our findings demonstrate for the first time a relationship between structural and functional connectivity within the cortico-NBM network and motivate future studies aimed at disentangling the relative contribution of the identified networks in both normal neurophysiological functioning and neurological disease.

## Funding

A.O. is supported by an NIHR Academic Clinical Lectureship and an Academy of Medical Sciences Starter Grant. The Wellcome Centre for Human Neuroimaging is supported by core funding from Wellcome [203147/Z/16/Z]. The UK MEG community was supported by Medical Research Council grant MK/K005464/1. P.B. is supported by the Medical Research Council (MC_UU_12024/5). L.Za. is supported by the NIH/NINDS grant number: 2RF1NS023945-28. T.F. has received grants from the NIHR, Michael J Fox Foundation, Cure Parkinson’s Trust, Innovate UK, John Black Charitable Foundation, Janet Owens Fellowship & Defeat MSA. L.Zr and H.A. are supported by a grant from the Brain Research Trust (157806) and the National Institute for Health Research University College London Hospitals Biomedical Research Centre. The Unit of Functional Neurosurgery is supported by the Parkinson's Appeal and the Sainsbury Monument Trust.

## Competing interests

J.G. reports honoraria from Medtronic, UCB Pharmaceuticals, Britannia Pharmaceuticals and Bial. L.Zr. and M.H. report honoraria and travel expenses from Medtronic and Boston Scientific for speaking at meetings. T.F. has received honoraria for talks sponsored by Bial, Profie Pharma, Boston Scientific and has served on advisory boards for Living Cell Technologies and Neurocrine & Handl therapeutics. P.B. is a consultant for Medtronic. All other authors report no competing interests.

## Abbreviations

DBS: deep brain stimulation
DLB: Parkinson’s disease dementia
GPe/i: globus pallidus pars externa/interna
LFP: local field potentials
MEG: magnetoencephalography
NBM: nucleus basalis of Meynert
PDD: Parkinson’s disease dementia
